# Travis County Medical Society

**Published:** 1887-04

**Authors:** 


Travis County Medical Society; At the regular monthly meeting of T. C. Med. Soc. April 7th, seven new members were added: to-wit Drs. R. S. Gregg and W. T. Richmond, of Manor,, Dr. F. R. Martin of Kyle, Dr. H. H. Thorpe of Liberty Hill, Dr. J. W. Hamilton of Hornsby's, Dr. S. B. Hill of Webberville and Dr. J. O. Lewright of Austin. Two of these gentlemen are members of the State Association, the other five will join at the meeting.

